# Prevention is less expensive than treatment in chronic kidney disease: a descriptive analysis

**DOI:** 10.1590/2175-8239-JBN-2025-0122en

**Published:** 2025-12-05

**Authors:** Paulo Roberto Alcalde, Danilo Euclides Fernandes, Gianna Mastroianni Kirsztajn

**Affiliations:** 1 Universidade Federal de São Paulo, Departamento de Medicina, Divisão de Nefrologia, São Paulo, SP, Brazil.

**Keywords:** Health Expenditures, Primary Health Care, Public Health, Renal Insufficiency, Primary Care, Community Health

## Abstract

**Introduction:**

The prevalence of chronic kidney disease (CKD) is increasing worldwide, leading to great concern regarding costs, particularly in underdeveloped or developing countries. Estimating the costs of different CKD management approaches is necessary for planning public policies.

**Objectives:**

To evaluate the costs of renal replacement therapy (RRT) and non-RRT treatment and to propose the screening for CKD in the Brazilian Health Care System (SUS).

**Methods:**

This descriptive analysis was based on information from the Brazilian Health Ministry database, DATASUS (www.datasus.saude.gov.br), and scheduled payments approved by the SUS, including complementary exams related to CKD screening, diagnosis and treatment, recommended assessments by stages of CKD, and health professionals’ salaries.

**Results:**

Values (in US$) of the probable annual costs for CKD since diagnosis, followed by different CKD stages, and the RRT phase are: first evaluation, 5.00; first evaluation of individuals at risk, 12.03; CKD stage 1, 5.00; 2, 7.36; 3, 53.32; 3B, 36.85; 4, 129.51; 5–non dialytic, 183.73; 5D–peritoneal dialysis, 3,539.10; and stage 5D–hemodialysis (7,925.87).

**Conclusion:**

The cost of screening (early diagnosis) for CKD or initial assessment of CKD stage 1 for one patient, including complementary exams and medical appointments, is US$ 5.00 (based on standard SUS reimbursement values). The costs of 1 year of treatment for a single patient with stage 5D CKD on hemodialysis could cover care for 1,584 with stage 1 CKD or 61 patients with stage 4 CKD. Our data indicates a clear economic advantage of early CKD diagnosis for public health.

## Introduction

Chronic kidney disease (CKD) is defined as the presence of renal injury and/or decrease of glomerular filtration rate (GFR) < 60 mL/min/1.73 m^2^ of body surface area for a minimum of 3 months, regardless of the underlying cause1. Renal injury can be established, for instance, by the detection of albuminuria even in the absence of impaired GFR.

The treatment approach adopted after diagnosis of CKD depends on the stage of CKD at the moment the patient enters the health system, among other factors. The prognosis depends on the causes of CKD. Some may benefit from conservative treatment and others may need immediate renal replacement therapy (RRT). Thus, the final cost varies according to the complexity of the treatment approach. Besides, it is known that late diagnosis and consequent late referral (shortly before dialysis needs to be started) leads to a higher mortality risk during maintenance dialysis^2,3^, which may have been preventable and less costly^2^.

Epidemiological data from the United States suggest that 200 patients with stage 3 or 4 CKD and 5,000 patients with stages 1 or 2 CKD are equivalent in cost to a single patient with end-stage renal disease (ESRD). However, the actual numbers of patients in each CKD stage are unknown in most countries; consequently, available data on CKD are generally based on limited information about the advanced stages of CKD, which represent only a small proportion of the overall CKD population^4^.

CKD affects approximately 11% of the United States’ population^4^. Studies from Australia, Europe, and Japan describe a prevalence of 6 to 16% for different CKD stages. Populational surveys based on the detection of microalbuminuria have shown a frequency of 6 to 7%^5^. Similar findings were observed in Brazil^6^.

Considering the complexity of the health care for CKD, the negative impact of CKD on patients and their families and the elevated costs of RRT, CKD prevention may be the most cost-effective approach^1^. However, screening for CKD in the general population and not just among high-risk individuals is not a consensus worldwide^7^.

The present study aimed to demonstrate, based on the costs of each CKD approach, that prevention (early diagnosis) is the best alternative to reduce the burden that CKD treatment represents for the public health system. In Brazil, the government-funded Unified Healthcare System (SUS) provides health care free of charge for the entire population.

## Methods

Methods are briefly presented here and described in detail in the supplementary material.

Data were collected from the Brazilian Health System database DATASUS^7^ (www.datasus.saude.gov.br) through access to the generic data application (TABNET) in the Healthcare item of the following groups: Hospital Production, SUS Hospital Information System (SIH/SUS) and Outpatient Production, SUS Outpatient Information System (SIA/SUS), and the SUS Procedures, Medications, Orthoses, Prostheses, and Materials Table Management System – SIGTAP, which were used as reference for calculating the costs of the recommendations of the Guidelines for CKD of the Brazilian Health System^8^, such as establishing the number of complementary exams and the type and number of visits per year.

The amounts reimbursed by the SUS were used as a basis for estimating annual costs for RRT compared to that of renoprotective conservative therapy, as well as for prevention, using a simplified proposal of screening for CKD, i.e. the first assessment by the general practitioner consisting of measuring serum creatinine (estimated GFR) and urinalysis.

We converted all the values in Brazilian reals (R$) to American dollars (US$) at the time this study was conducted.

This study protocol was approved by the local Institutional Ethics Committee (approval number 786.188).

## Results

**Figure 1.**
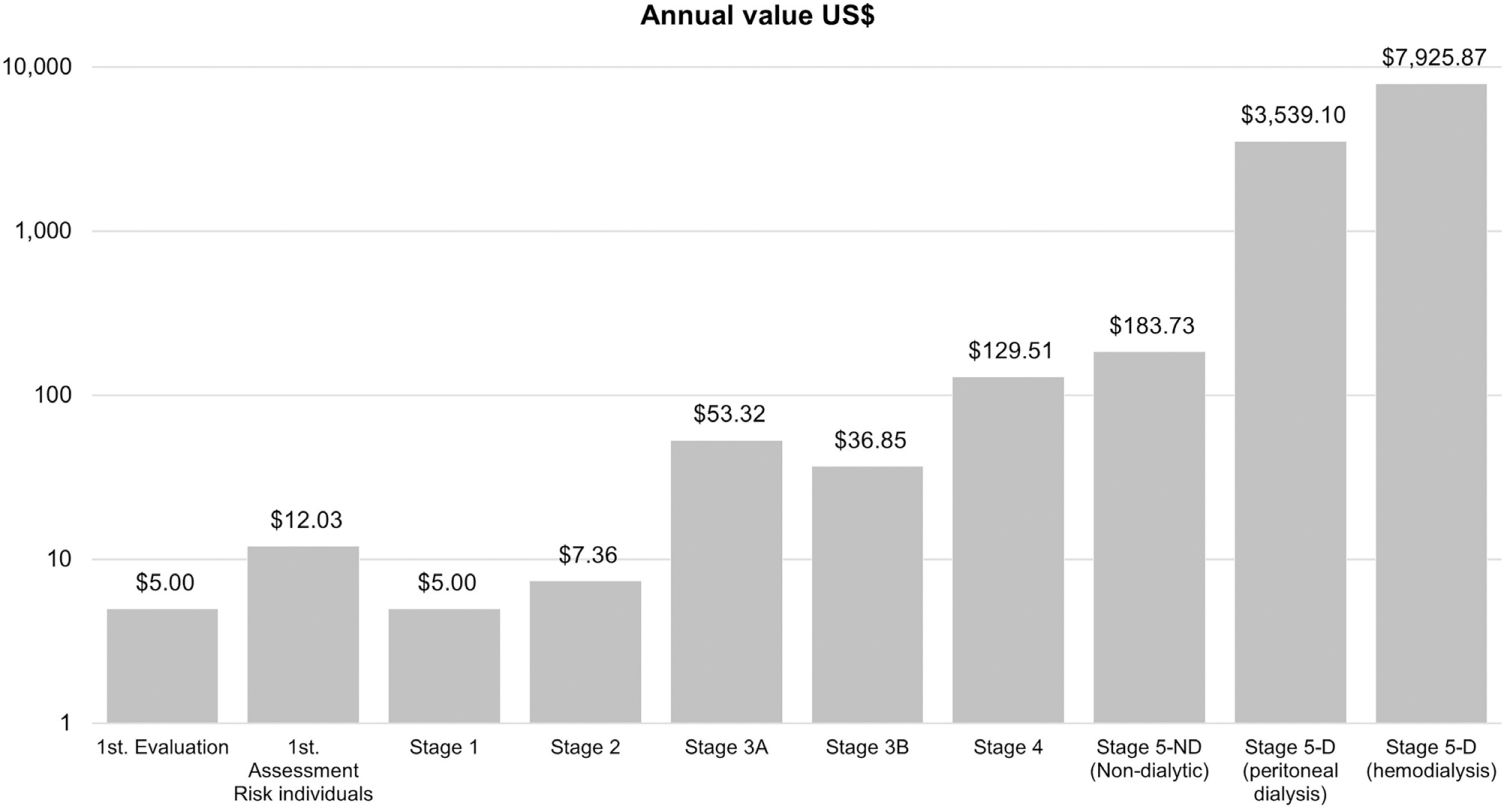
Annual values (in US$) paid by the Brazilian Unified Healthcare System for each type of assessment and/or chronic kidney disease stage.

Initially, we presented CKD costs by the time of diagnosis, in each CKD stage, and in ESRD, including RRT. Table 1 and Figure 1 show the annual values that were paid (in US$) by the SUS to healthcare workers and complementary exams recommended for each CKD stage according to Brazilian Ministry of Health Guidelines and procedures, when necessary.

**Table 1:** Unified healthcare system reimbursement values (in united states dollars, us$) of healthcare workers’ fees and complementary exams to be performed in ckd stages according to the brazilian ckd guidelines

Assessment/Stages	Professional involved	Exams	Annual value in US$
First assessment	General practitioner	*Annual:* GFR, urinalysis	5.00
First assessment in individuals at risk	General practitioner	*Annual:* GFR, urinalysis. Ultrasonography of kidneys and urinary tract	12.03
Stage 1	General practitioner	*Annual:* GFR, urinalysis.	5.00
Stage 2	General practitioner	*Annual:* GFR, urinalysis, and RAC	7.36
Stage 3A	General practitioner	*Annual:* GFR, urinalysis, RAC, potassium, phosphorus, and PTH. Serology for hepatitis B (HbsAg, Anti-HBc IgG and Anti-HBs)	53.32
Stage 3B	General practitioner	*Every 6 months:* GFR, urinalysis, RAC, and potassium. *Annual:* calcium, phosphorous, PTH and total proteins and fractions, hematocrit, and hemoglobin, ferritin, IST	36.85
Stage 4	Multi-professional team: nephrologist, nurse, nutritionist, psychologist, social worker	*Quarterly:* creatinine, urea, calcium, HemoCue, and hemoglobin, ferritin, and IST in patients with anemia and potassium. Midterms: PTH, alkaline phosphatase, venous gas or alkaline reserve, total proteins and fractions, and RAC. *Annual:* Anti-HBs,	129.51
Stage 5-ND (Non-dialytic)	Multi-professional team: nephrologist, nurse, nutritionist, psychologist, social worker	*Monthly:* urea, creatinine, calcium, phosphorus, potassium, hemoglobin, and hematocrit. *Quarterly:* total proteins and fractions, ferritin, IST, alkaline phosphatase, PTH, and venous gasometry. *Every 6 months:* vitamin D; Annual exams: anti-HBS, anti-HCV, HBsAg, and HIV	183.73
Stage 5D (peritoneal dialysis)	Multi-professional team: nephrologist, nurse, nutritionist, psychologist, social worker	*Monthly:* hematocrit, hemoglobin, urea before and after the hemodialysis session, sodium, potassium, calcium, phosphorus, TGP, blood glucose for patients with diabetes, and creatinine during the first year. *Quarterly:* complete blood count, saturation index of transferrin, dosage of ferritin, phosphatase alkaline, PTH, total protein and fractions and glycated hemoglobin for patients with diabetes. *Every 6 months:* vitamin D and anti-HBs. Annual exams: total cholesterol, triglycerides, and serum aluminum, glucose, TSH, T4, dosage of antibodies to HIV, chest x-rays (AP and lateral), kidney, and urinary tract ultrasound, electrocardiogram.	3,539.10
Stage 5D (hemodialysis)	Multi-professional team: nephrologist, nurse, nutritionist, psychologist, social worker	*Monthly:* hematocrit, hemoglobin, urea before and after the hemodialysis session, sodium, potassium, calcium, phosphorus, GPT, blood glucose for patients with diabetes and creatinine during the first year. TGP, AntiHBc IgM, HbsAg and AntiHCV. *Quarterly:* complete blood count, IST, alkaline phosphatase, PTH, ferritin, total proteins and fractions, and glycated hemoglobin for patients with diabetes. *Every 6 months:* vitamin D and susceptible patients, antiHBs, total AntiHBC or IgG, HBsAg, AntiHCV. *Annual:* total cholesterol, triglycerides, and serum aluminum, glucose, TSH, T4, antiHIV, chest x-rays (AP and lateral); renal and urinary tract ultrasound, electrocardiogram. *Eventual:* blood culture in case of suspected infection of the bloodstream and test of desferal in case of suspected aluminum poisoning.	7,925.87

Abbreviations – CKD: chronic kidney disease; RAC: urinary albumin/creatinine ratio; GFR: glomerular filtration rate; IST: transferrin saturation index; PTH: parathormone; GPT: glutamic-pyruvic transaminase; TSH: thyroid-stimulating hormone; T4: thyroxine; AP: anteroposterior; HBcAg: hepatitis B core antigen; HbsAg: hepatitis B virus surface antigen; IgG: immunoglobulin G; HCV: hepatitis C virus; HIV: human immunodeficiency virus.

The comparative analyses between costs of “preventive” screening for CKD and of other management/treatment approaches in different stages of CKD are shown in Table 2. The percent increase of costs in different stages of CKD is also presented.

**Table 2:** Percentage difference in costs between stages of chronic kidney disease from prevention to renal replacement therapy

Standard values paid for CKD management approaches (in US$)		
Cost 1 (US$)	Cost 2 (US$)	% Difference
First assessment (prevention) and stage 1	Stage 4	
5.00	129.51	2,490.20
First assessment (prevention) and stage 1	Stage 5D in peritoneal dialysis	
5.00	3,539.10	70,682.00
First assessment (prevention) and stage 1	Stage 5D in hemodialysis	
5.00	7,925.87	158,417.40
First assessment (prevention) and stage 1	Kidney transplant (deceased donor organ)	
5.00	8,027.51	160,450.20
Stage 4	Stage 5D in peritoneal dialysis	
129.51	3,539.10	2,632.68
Stage 4	Stage 5D in hemodialysis	
129.51	7,925.87	6,019.89
Stage 4	Kidney transplant (deceased donor organ)	
129.51	8,027.51	6,098.37

Notes – % difference: [cost2 – cost1]/cost1 × 100); percent increase between the values of the 1st column and the 2nd column. US$: United States dollars. Values are exact numbers.

## Discussion

CKD is increasing worldwide, causing great concern regarding health costs. RRT is essential for sustaining the lives of patients with ESRD; however its high cost can be an obstacle, particularly in developing countries. Therefore, cost evaluation of different CKD management approaches may be necessary for planning and defining public health policies.

We demonstrate that the cost-benefit of preventing CKD overcomes treatment costs in later stages of CKD in Brazil and possibly in other countries, especially developing ones. Although it seems obvious, prevention is still not widely recommended worldwide as a public health strategy.

Table 2 shows remarkable cost differences between “preventive” and other approaches in CKD. Based on the Brazilian guidelines for CKD^9^, we designed a table to better compare the healthcare costs from early to advanced stages of CKD, as well as preventive approaches, when no manifestation of CKD is detected. This was achieved by calculating the annual values of appointments and the minimal recommended complementary medical exams needed for clinical management. The costs of both, first evaluation and stage 1 CKD treatment, were considered equivalent. In that early stage, only the groups at risk presented significant differences. Clinically, such resources can detect CKD and are extremely important because they allow the evaluation of GFR values, which is essential for categorizing patients into stages of CKD and determining the appropriate management of that disease stage.

In the present study, which utilized data from standardized payments performed by the SUS for staff and procedures, we observed that healthcare costs markedly increased from stage 3 CKD onwards, with an increase from stage 3 to 4 of 143%, from stage 4 to 5 (in peritoneal dialysis) of 2,633%, and from stage 5 on peritoneal dialysis to 5 on hemodialysis) of 124%. The rise from stage 1 to renal transplantation reached 160,450%.

The annual cost of one individual with stage 5 CKD on hemodialysis can conservatively treat 61 patients in stage 4 or even 1,584 patients in stage 1 CKD. The annual cost of a patient progressing from stage 4 to 5 on hemodialysis is US$ 483,618.64 and of a patient in stage 1 progressing to stage 5 on hemodialysis is US$ 12,558,228.28. Thus, if we were able to stop 1,584 patients in stage 1 from progressing to stage 5, we would be saving approximately US$ 12 million every year. Certainly, it is impossible to predict how long a patient will be in stage 1, but regular follow-up and satisfactory management of the CKD may delay ESRD and the need for RRT. The saving is evident, regardless eventual additional costs related to ESRD, such as disability, retirement, and worsening of quality of life.

In the United States, it was estimated that 11% of adults have CKD^2^. Based on data from 2010 Brazilian populational survey^10^, in which adults corresponded to 67%, an estimated 14 million Brazilians would have different stages of CKD.

There is no agreement on populational screening for CKD^11-14^. In fact, different studies indicate that it only would be cost-effective among specific high-risk groups^11^. Some studies suggest that those groups should include individuals who are >55 years of age^11^, or those with hypertension or diabetes^7^. For such groups, some authors recommend screening CKD through an annual albuminuria investigation^12^. Others claim that the assessment of a combination of albuminuria, serum creatinine, and eGFR would be advisable^13^, and this combination of tests is currently recommended for early identification of CKD and risk stratification. Nevertheless, basic screening for CKD is not yet routinely performed, as can be deduced from recent analysis of a nationwide laboratory database in Brazil showing that a significant proportion of individuals with a serum creatinine test lack an accompanying albuminuria measurement^15^.

Our study has limitations such as the fixed SUS reimbursement values (which may underestimate real-world costs), the absence of clinical outcome data, and the inability to estimate indirect costs (e.g., hospitalization, productivity loss). Besides, although showing that early-stage treatment is cheaper, this does not imply that screening is a better strategy, i.e. this finding alone does not confirm that population-wide screening is cost-effective.

## Conclusions

The cost of screening (early diagnosis) for CKD or initial assessment of stage 1 CKD for one patient, including complementary exams and medical appointments, is US$ 5.00 (standard values paid by SUS). The annual cost of treating one patient with 5D CKD on hemodialysis could possibly treat 1,584 and 61 patients with stages 1 and 4 CKD, respectively, for the same period.

Our findings indicate an unequivocal public health economic benefit of early diagnosis of CKD in a developing country such as Brazil, whose population is predominantly assisted by a public health care system.

## Data Availability

If necessary, data is available for consultation upon request to the corresponding author.

## Supplementary Material

The following online material is available for this article:

## References

[1] Stevens PE, Ahmed SB, Carrero JJ, Foster B, Francis A, Hall RK (2024). Kidney Disease: Improving Global Outcomes (KDIGO) CKD Work Group. KDIGO 2024 Clinical Practice Guideline for the Evaluation and Management of Chronic Kidney Disease.. Kidney Int..

[2] Ratcliffe PJ, Phillips RE, Oliver DO (1984). Late referral for maintenance dialysis.. Br Med J (Clin Res Ed)..

[3] Sesso R, Belasco AG (1996). Late diagnosis of chronic renal failure and mortality on maintenance dialysis.. Nephrol Dial Transplant..

[4] Coresh J, Astor BC, Greene T, Eknoyan G, Levey AS (2003). Prevalence of chronic kidney disease and decreased kidney function in the adult US population: Third National Health and Nutrition Examination Survey.. Am J Kidney Dis..

[5] Meguid El Nahas A, Bello AK (2005). Chronic kidney disease: the global challenge.. Lancet..

[6] de Lima AO, Kesrouani S, Gomes RA, Cruz J, Mastroianni-Kirsztajn G (2012). Population screening for chronic kidney disease: a survey involving 38,721 Brazilians.. Nephrol Dial Transplant..

[7] Komenda P, Ferguson TW, Macdonald K, Rigatto C, Koolage C, Sood MM (2014). Cost-effectiveness of primary screening for CKD: a systematic review.. Am J Kidney Dis..

[8] DATASUS. (2025). Portal da Saúde – O DATASUS [Internet]..

[9] Brasil. (2014). Ministério da Saúde. Secretaria de Atenção à Saúde. Departamento de Atenção Especializada e Temática. Diretrizes Clínicas para o Cuidado ao paciente com Doença Renal Crônica – DRC no Sistema Único de Saúde [Internet]..

[10] Instituto Brasileiro de Geografia e Estatística. (2010). Censo demográfico [Internet]..

[11] The Bmj. (2006). Is screening for chronic kidney disease cost effective?. BMJ.

[12] Dirks JH, De Zeeuw D, Agarwal SK, Atkins RC, Correa-Rotter R, D’Amico G (2005). Prevention of chronic kidney and vascular disease: toward global health equity—the Bellagio 2004 Declaration.. Kidney Int..

[13] Middleton RJ, Foley RN, Hegarty J, Cheung CM, McElduff P, Gibson JM (2006). The unrecognized prevalence of chronic kidney disease in diabetes.. Nephrol Dial Transplant..

[14] Yarnoff BO, Hoerger TJ, Simpson SK, Leib A, Burrows NR, Shrestha SS (2017). The cost-effectiveness of using chronic kidney disease risk scores to screen for early-stage chronic kidney disease.. BMC Nephrol..

[15] Guedes M, Dias PT, Réa RR, Calice-Silva V, Lopes M, Brandão AA (2024). Patterns of kidney function and risk assessment in a nationwide laboratory database: the Brazilian CHECK-CKD study.. BMC Nephrol..

