# Supplementary material for: Prevention is less expensive than treatment in chronic kidney disease: a descriptive analysis
Source: J Bras Nefrol. 2025 Dec 5;48(1):e20250122. doi: 10.1590/2175-8239-JBN-2025-0122en (PMC12683897; doi:10.1590/2175-8239-JBN-2025-0122en)
Supplement: METHODS [file 2175-8239-jbn-48-1-e20250122-suppl1.pdf]

*Braz. J. Nephrol.*

https://doi.org/10.1590/2175-8239-JBN-2025-0122en

# Supplementary Material to "Prevention is less expensive than treatment in chronic kidney disease: a descriptive analysis"

## METHODS

This is a descriptive study that used data collected from the Ministry of Health's Data System – DATASUS (www.datasus.saude.gov.br), through access to information in the generic data application (TABNET), in the Healthcare item in the groups: Hospital Production, SUS Hospital Information System (SIH/SUS) and Outpatient Production, SUS Outpatient Information System (SIA/SUS), and the SUS Procedures, Medications, Orthoses, Prostheses, and Materials Table Management System – SIGTAP.

The amounts paid by the SUS were evaluated as a basis for estimating annual expenditures for CKD treatment in Brazil and hospitalizations for CKD and CKD-associated diseases, as well as tests related to their detection and treatment.

For all analyses reported here, annual costs for the years 2013 to 2015, as published by DATASUS, were included.

To determine the costs of healthcare professionals' fees and complementary tests to be used in the stages of CKD based on the Clinical Guidelines for the care of patients with chronic kidney disease (CKD) in the Unified Health System (SUS), we accessed the SUS Procedures, Medications, Orthoses, Prostheses, and Materials Table Management System (SIGTAP) at the website: www.sigtap.datasus.gov.br, then selected Unified Table and consulted procedures.

The tests whose data were obtained through the "procedure" field are coded according to the SUS Table, as well as dialysis and kidney transplant data.

For example, the main tests used and their codes corresponded to: creatinine – 202010317; creatinine clearance – 202050025; urea – 202010694; analysis of physical characteristics, elements, and urine sediment – 202050017; protein – 202050114; culture for bacterial identification – 202080080; kidney puncture biopsy – 201010437; urinary tract ultrasound – 205020054; glomerular filtration rate – 208040080.

We converted all the costs in Brazilian currency (reais, R$) to American dollars (US$) by the time this study was conducted, with the aim of having costs translated into a reference currency, stable and whose value is widely known in different regions of the world.
