# Supplementary material for: Prevention is less expensive than treatment in chronic kidney disease: a descriptive analysis
Source: J Bras Nefrol. 2025 Dec 5;48(1):e20250122. doi: 10.1590/2175-8239-JBN-2025-0122en (PMC12683897; doi:10.1590/2175-8239-JBN-2025-0122en)
Supplement: Translation

# Translation: A prevenção é menos onerosa que o tratamento da doença renal crônica: análise descritiva

**DOI:** 10.1590/2175-8239-JBN-2025-0122pt

**Authors:** Paulo Roberto Alcalde, Danilo Euclides Fernandes, Gianna Mastroianni Kirsztajn

**Affiliations:** 1 Universidade Federal de São Paulo, Departamento de Medicina, Divisão de Nefrologia, São Paulo, SP, Brasil.

**Keywords:** Despesas Com Saúde, Atenção Primária À Saúde, Saúde Pública, Insuficiência Renal, Cuidados Primários, Saúde Comunitária

## Abstract

**Introdução:**

A prevalência da doença renal crônica (DRC) vem aumentando globalmente, gerando grande preocupação quanto aos custos, especialmente em países subdesenvolvidos ou em desenvolvimento. É necessário estimar custos das diferentes abordagens de manejo da DRC para planejar políticas públicas.

**Objetivos:**

Avaliar custos da terapia renal substitutiva (TRS) e tratamentos não relacionados à TRS. Propor triagem da DRC no Sistema Único de Saúde (SUS).

**Métodos:**

Análise descritiva baseada no banco de dados do Ministério da Saúde do Brasil, DATASUS (www.datasus.saude.gov.br), e pagamentos programados aprovados pelo SUS, incluindo exames complementares relacionados à triagem, diagnóstico e tratamento da DRC, avaliações recomendadas por estágios da doença e salários dos profissionais de saúde.

**Resultados:**

Os valores (em US$) dos custos anuais prováveis para DRC desde o diagnóstico, seguidos pelos diferentes estágios da doença e fases da TRS, são: primeira avaliação: 5,00; primeira avaliação - indivíduos em risco: 12,03; DRC estágio 1: 5,00; 2: 7,36; 3: 53,32; 3B: 36,85; 4: 129,51; 5 – não dialítico: 183,73; 5D – diálise peritoneal: 3.539,10; e estágio 5D – hemodiálise: 7.925,87.

**Conclusão:**

O custo da triagem (diagnóstico precoce) para DRC ou avaliação inicial do estágio 1 em um paciente, incluindo exames complementares e consultas médicas, é de US$ 5,00 (baseado nos valores padrão de reembolso do SUS). Os custos de um ano de tratamento para um único paciente com DRC-5D em hemodiálise poderiam cobrir os cuidados de 1.584 pacientes no estágio 1 ou 61 pacientes no estágio 4 da DRC. Nossos dados indicam clara vantagem econômica do diagnóstico precoce para a saúde pública.

## Introdução

A doença renal crônica (DRC) é definida pela presença de lesão renal e/ou pela redução da taxa de filtração glomerular (TFG) para valores inferiores a 60 mL/min/1,73 m^2^ de área de superfície corporal, mantida por um período mínimo de 3 meses, independentemente da causa subjacente^1^. A lesão renal pode ser estabelecida, por exemplo, pela detecção de albuminúria, mesmo na ausência de comprometimento da TFG.

A abordagem terapêutica adotada após o diagnóstico de DRC depende do estágio da doença no momento em que o paciente ingressa no sistema de saúde, entre outros fatores. O prognóstico está relacionado às causas da DRC: alguns pacientes podem se beneficiar do tratamento conservador, enquanto outros podem necessitar de TRS imediata. Dessa forma, o custo final varia conforme a complexidade da abordagem terapêutica. Além disso, sabe-se que o diagnóstico e o encaminhamento tardios (pouco antes do início da diálise) levam a um risco aumentado de mortalidade durante a diálise de manutenção^2,3^, situação que pode ser evitada, determinando custos menores^2^.

Dados epidemiológicos provenientes dos Estados Unidos sugerem que 200 pacientes com DRC em estágios 3 ou 4 e 5.000 pacientes em estágios 1 ou 2 equivalem, em termos de custo, a um único paciente com doença renal em estágio terminal (DRET). No entanto, os números reais de pacientes em cada estágio da DRC são desconhecidos na maioria dos países; consequentemente, os dados disponíveis sobre a DRC geralmente se baseiam em informações limitadas sobre os estágios avançados da doença, que representam apenas uma pequena proporção da população total acometida^4^.

A DRC afeta aproximadamente 11% da população dos Estados Unidos^4^. Estudos realizados na Austrália, Europa e Japão descrevem uma prevalência de 6 a 16% para os diferentes estágios da doença. Pesquisas populacionais com base na detecção de microalbuminúria demonstraram uma frequência de 6 a 7%^5^. Achados semelhantes foram observados no Brasil^6^.

Considerando a complexidade dos cuidados em saúde relacionados à DRC, o impacto negativo da doença nos pacientes e suas famílias, bem como os elevados custos da TRS, a prevenção pode constituir a abordagem mais eficaz em termos de custo-benefício^1^. No entanto, a realização de exames de triagem para DRC na população em geral, e não apenas entre indivíduos de alto risco, ainda não é um consenso em todo o mundo^7^.

O presente estudo teve como objetivo demonstrar, com base nos custos de cada abordagem da DRC, que a prevenção (diagnóstico precoce) constitui a melhor alternativa para reduzir o ônus que o tratamento da doença representa para o sistema público de saúde. No Brasil, o Sistema Único de Saúde (SUS), financiado pelo governo, oferece assistência médica gratuita a toda a população.

## Métodos

Os métodos são brevemente apresentados nesta seção e descritos em detalhes no material suplementar.

Os dados foram coletados do banco de dados do Sistema Único de Saúde (SUS) DATASUS^7^ (www.datasus.saude.gov.br) por meio do acesso ao aplicativo de dados genéricos (TABNET), no item Assistência à Saúde, dos seguintes grupos: Produção Hospitalar, Sistema de Informações Hospitalares do SUS (SIH/SUS) e Produção Ambulatorial, Sistema de Informações Ambulatoriais do SUS (SIA/SUS), além do Sistema de Gerenciamento da Tabela de Procedimentos, Medicamentos, Órteses, Próteses e Materiais – SIGTAP. Esses dados foram utilizados como referência para o cálculo dos custos estabelecidos pelas recomendações das Diretrizes para DRC do Sistema Único de Saúde^8^, tais como a definição do número de exames complementares e o tipo e número de consultas por ano.

Os valores de reembolso do SUS foram utilizados como base para a estimativa dos custos anuais da TRS, em comparação aos do tratamento conservador renoprotetor, bem como para a prevenção, utilizando uma proposta simplificada de triagem para DRC, ou seja, a primeira avaliação realizada pelo clínico geral, consistindo na medição da creatinina sérica (TFG estimada) e no exame de urina.

Todos os valores em reais (R$), foram convertidos em dólares estadunidenses (US$) na data em que este estudo foi realizado.

O protocolo deste estudo foi aprovado pelo Comitê de Ética Institucional local (número de aprovação 786.188).

## Resultados

Inicialmente, apresentamos os custos da DRC no momento do diagnóstico, em cada estágio da doença e na DRET, incluindo a TRS. A Tabela 1 e a Figura 1 mostram os valores anuais pagos (em US$) pelo SUS aos profissionais de saúde e os exames complementares recomendados para cada estágio da DRC, de acordo com as Diretrizes e procedimentos do Ministério da Saúde do Brasil, quando necessário.

**Tabela 1:** Valores de reembolso do sistema único de saúde (em dólares estadunidenses, us$) referentes aos honorários dos profissionais de saúde e exames complementares a serem realizados nos diferentes estágios da drc, conforme as diretrizes brasileiras para drc

Avaliação/ Estágios	Profissional envolvido	Exames	Valor anual em US$
Primeira avaliação	Clínico geral	*Anual:* TFG, urinálise.	5,00
Primeira avaliação em indivíduos de risco	Clínico geral	*Anual:* TFG, urinálise. Ultrassonografia dos rins e do trato urinário.	12,03
Estágio 1	Clínico geral	*Anual:* TFG, urinálise.	5,00
Estágio 2	Clínico geral	*Anual:* TFG, urinálise e RAC.	7,36
Estágio 3A	Clínico geral	*Anual:* TFG, urinálise, RAC, potássio, fósforo e PTH. Sorologia para hepatite B (HbsAg, Anti-HBc IgG e Anti-HBs).	53,32
Estágio 3B	Clínico geral	*A cada 6 meses:* TFG, urinálise, RAC e potássio. *Anual:* cálcio, fósforo, PTH e proteínas totais e frações, hematócrito e hemoglobina, ferritina, IST.	36,85
Estágio 4	Equipe multiprofissional: nefrologista, enfermeiro, nutricionista, psicólogo, assistente social	*Trimestral:* creatinina, ureia, cálcio, HemoCue e hemoglobina, ferritina e IST em pacientes com anemia e potássio. Exames intermediários: PTH, fosfatase alcalina, gás venoso ou reserva alcalina, proteínas totais e frações e RAC. *Anual:* Anti-HBs.	129,51
Estágio 5-ND (Não Dialítico)	Equipe multiprofissional: nefrologista, enfermeiro, nutricionista, psicólogo, assistente social	*Mensal:* ureia, creatinina, cálcio, fósforo, potássio, hemoglobina e hematócrito. *Trimestral:* proteínas totais e frações, ferritina, IST, fosfatase alcalina, PTH e gasometria venosa. *A cada 6 meses:* vitamina D; Exames anuais: anti-HBS, anti-HCV, HBsAg e HIV.	183,73
Estágio 5D (Diálise Peritoneal)	Equipe multiprofissional: nefrologista, enfermeiro, nutricionista, psicólogo, assistente social	*Mensal:* hematócrito, hemoglobina, ureia antes e após a sessão de hemodiálise, sódio, potássio, cálcio, fósforo, TGP, glicemia para pacientes com diabetes e creatinina durante o primeiro ano. *Trimestral:* Hemograma completo, índice de saturação da transferrina, dosagem de ferritina, fosfatase alcalina, PTH, proteína total e frações e hemoglobina glicada para pacientes com diabetes. *A cada 6 meses:* vitamina D e anti-HBs. Exames anuais: colesterol total, triglicérides e alumínio sérico, glicose, TSH, T4, dosagem de anticorpos para HIV, radiografias de tórax (PA e lateral), ultrassonografia dos rins e do trato urinário, eletrocardiograma.	3.539,10
Estágio 5D (Hemodiálise)	Equipe multiprofissional: nefrologista, enfermeiro, nutricionista, psicólogo, assistente social	*Mensal:* hematócrito, hemoglobina, ureia antes e após a sessão de hemodiálise, sódio, potássio, cálcio, fósforo, TGP, glicemia para pacientes com diabetes e creatinina durante o primeiro ano. TGP, AntiHBc IgM, HbsAg e AntiHCV. *Trimestral:* hemograma completo, IST, fosfatase alcalina, PTH, ferritina, proteínas totais e frações e hemoglobina glicada para pacientes com diabetes. *A cada 6 meses:* vitamina D e pacientes suscetíveis, antiHBs, AntiHBC total ou IgG, HBsAg, AntiHCV. *Anual:* colesterol total, triglicérides e alumínio sérico, glicose, TSH, T4, anti-HIV, radiografias de tórax (PA e lateral); ultrassonografia dos rins e do trato urinário, eletrocardiograma. *Eventual:* hemocultura em caso de suspeita de infecção da corrente sanguínea e teste à desferroxamina em caso de suspeita de intoxicação por alumínio.	7.925,87

Abreviaturas – DRC: doença renal crônica; RAC: relação albumina/creatinina urinária; TFG: taxa de filtração glomerular; IST: índice de saturação da transferrina; PTH: paratormônio; TGP: transaminase glutâmico-pirúvica; TSH: hormônio estimulante da tireoide; T4: tiroxina; PA: póstero-anterior; HBcAg: antígeno core do vírus da hepatite B; HbsAg: antígeno de superfície do vírus da hepatite B; IgG: imunoglobulina G; HCV: vírus da hepatite C; HIV: vírus da imunodeficiência humana.

**Figura 1.** Valores anuais (em US$) pagos pelo Sistema Único de Saúde brasileiro para cada tipo de avaliação e/ou estágio da doença renal crônica.

As análises comparativas entre os custos da triagem “preventiva” para DRC e de outras abordagens de manejo/tratamento em diferentes estágios da doença são apresentadas na Tabela 2. O aumento percentual dos custos nos diferentes estágios também é apresentado.

**Tabela 2:** Diferença percentual nos custos entre os estágios da doença renal crônica, desde a prevenção até a terapia renal substitutiva

Valores padrão pagos para as abordagens de manejo da DRC (em US$).		
Custo 1 (US$)	Custo 2 (US$)	% Diferença
Primeira avaliação (prevenção) e estágio 1	Estágio 4	
5,00	129,51	2.490,20
Primeira avaliação (prevenção) e estágio 1	Estágio 5D em diálise peritoneal	
5,00	3.539,10	70.682,00
Primeira avaliação (prevenção) e estágio 1	Estágio 5D em hemodiálise	
5,00	7.925,87	158.417,40
Primeira avaliação (prevenção) e estágio 1	Transplante renal (órgão de doador falecido)	
5,00	8.027,51	160.450,20
Estágio 4	Estágio 5D em diálise peritoneal	
129,51	3.539,10	2.632,68
Estágio 4	Estágio 5D em hemodiálise	
129,51	7.925,87	6.019,89
Estágio 4	Transplante renal (órgão de doador falecido)	
129,51	8.027,51	6.098,37

Notes – Diferença %: [custo2 – custo1] /custo1 × 100); aumento percentual entre os valores da 1ª coluna e da 2ª coluna. US$: Dólares estadunidenses. Os valores são números exatos.

## Discussão

A DRC vem apresentando um aumento global, causando grande preocupação em relação aos custos com a saúde. A TRS é essencial para a manutenção da vida de pacientes com DRET; entretanto, seu custo elevado pode representar um obstáculo, especialmente em países em desenvolvimento. Portanto, a avaliação dos custos relacionados às diferentes abordagens de manejo da DRC pode ser necessária para o planejamento e a definição de políticas públicas de saúde.

Demonstramos que a relação custo-benefício da prevenção da DRC supera os custos do tratamento em estágios mais avançados da doença no Brasil e, possivelmente, em outros países, especialmente aqueles em desenvolvimento. Embora pareça óbvio, a prevenção ainda não é amplamente recomendada em âmbito mundial como estratégia de saúde pública.

A Tabela 2 apresenta diferenças notáveis de custo entre as abordagens “preventivas” e as demais estratégias para a DRC. Com base nas diretrizes brasileiras para DRC^9^, elaboramos uma tabela com o objetivo de comparar, de maneira mais eficaz, os custos assistenciais desde os estágios iniciais até os avançados da doença, bem como das estratégias preventivas, quando não há manifestação detectável da DRC. Isso foi alcançado por meio do cálculo dos valores anuais referentes às consultas e aos exames médicos complementares mínimos recomendados para o manejo clínico. Os custos da primeira avaliação e do tratamento da DRC estágio 1 foram considerados equivalentes. Nessa fase inicial, somente os grupos de risco apresentaram diferenças significativas. Do ponto de vista clínico, esses recursos permitem a detecção da DRC e são de extrema importância, uma vez que possibilitam a avaliação dos valores da TFG, essenciais para a classificação dos pacientes em diferentes estágios da DRC e para a determinação do manejo adequado para cada um deles.

No presente estudo, que utilizou dados de pagamentos padronizados realizados pelo SUS para profissionais e procedimentos, observamos que os custos com saúde aumentaram de forma acentuada a partir do estágio 3 da DRC, com um aumento de 143% do estágio 3 para o 4, de 2.633% do estágio 4 para o 5 (em diálise peritoneal) e de 124% do estágio 5 em diálise peritoneal para o estágio 5 em hemodiálise. O aumento do estágio 1 para o transplante renal atingiu 160.450%.

O custo anual de um indivíduo com DRC estágio 5 em hemodiálise pode ser utilizado para o tratamento conservador de 61 pacientes em estágio 4 ou até mesmo 1.584 pacientes no estágio 1 da doença. O custo anual de um paciente que progride do estágio 4 para o 5 em hemodiálise é de US$ 483.618,64 e de um paciente no estágio 1 que progride para o estágio 5 em hemodiálise é de US$ 12.558.228,28. Assim, se fosse possível impedir que 1.584 pacientes no estágio 1 progredissem para o estágio 5, haveria uma economia aproximada de US$ 12 milhões por ano. Certamente, é impossível prever por quanto tempo um paciente permanecerá no estágio 1, mas o acompanhamento regular e o manejo adequado da DRC podem retardar a DRET e a necessidade de TRS. A economia é evidente, independentemente de eventuais custos adicionais relacionados à DRET, como invalidez, aposentadoria e piora da qualidade de vida.

Nos Estados Unidos, estima-se que 11% dos adultos apresentem DRC^2^. Com base nos dados do censo populacional brasileiro de 2010^10^, no qual os adultos correspondiam a 67% da população, estima-se que 14 milhões de brasileiros apresentem diferentes estágios da doença.

Não há consenso quanto à realização de triagem populacional para DRC^11-14^. De fato, diferentes estudos indicam que essa estratégia seria economicamente eficaz apenas para grupos específicos de alto risco^11^. Alguns relatos sugerem que esses grupos deveriam incluir indivíduos com idade superior a 55 anos^11^, ou aqueles com hipertensão ou diabetes^7^. Para tais grupos, certos autores recomendam a triagem da DRC através da investigação anual de albuminúria^12^. Outros afirmam que seria aconselhável a avaliação por meio de uma combinação das determinações de albuminúria, creatinina sérica e TFGe^13^, a qual é atualmente recomendada para a identificação precoce da DRC e a estratificação do risco. No entanto, a triagem básica para DRC ainda não é realizada de forma rotineira, como pode-se deduzir a partir de uma análise recente de banco de dados laboratorial de abrangência nacional no Brasil, que demonstra que uma proporção significativa de indivíduos com exame de creatinina sérica não apresenta medição concomitante de albuminúria^15^.

Nosso estudo apresenta limitações, tais como o uso de valores fixos de reembolso do SUS (que podem subestimar os custos reais), a ausência de dados de desfechos clínicos e a incapacidade de estimar custos indiretos (por exemplo, hospitalização, perda de produtividade). Além disso, embora demonstre que o tratamento em estágios iniciais é menos oneroso, isso não implica que a triagem seja uma estratégia mais eficaz - ou seja, esse achado, isoladamente, não confirma que a triagem em nível populacional seja uma estratégia custo-efetiva.

## Conclusões

O custo da triagem (diagnóstico precoce) para DRC ou da avaliação inicial de um paciente no estágio 1 da doença, incluindo exames complementares e consultas médicas, é de US$ 5,00 (valores padrão pagos pelo SUS). O custo anual do tratamento de um paciente com DRC em estágio 5D em hemodiálise poderia, possivelmente, cobrir o tratamento de 1.584 e 61 pacientes com DRC em estágios 1 e 4, respectivamente, no mesmo período.

Nossos achados indicam um benefício econômico inequívoco em termos de saúde pública do diagnóstico precoce da DRC em um país em desenvolvimento como o Brasil, cuja população é predominantemente assistida por um sistema público de saúde.

## Data Availability

Se necessário, os dados estão disponíveis para consulta mediante solicitação ao autor correspondente.

## Material Suplementar

O seguinte material online está disponível para o presente artigo:
